# Advances in the human skin microbiota and its roles in cutaneous diseases

**DOI:** 10.1186/s12934-022-01901-6

**Published:** 2022-08-29

**Authors:** Yudie Yang, Lingbo Qu, Ivan Mijakovic, Yongjun Wei

**Affiliations:** 1 School of Pharmaceutical Sciences, Zhengzhou University, Zhengzhou, 450051 China; 2 Laboratory of Synthetic Biology, Zhengzhou University, Zhengzhou, 450051 China; 3 College of Chemistry, Zhengzhou University, Zhengzhou, China; 4 Department of Biology and Biological Engineering, Chalmers University of Technology, Göteborg, Sweden; 5 The Novo Nordisk Foundation Center for Biosustainability, Technical University of Denmark, Lyngby, Denmark

**Keywords:** Skin, Microbiota, Commensal microbes, Synthetic biology, Omics technologies, Host-skin microbiota interaction, Cutaneous diseases, Acne

## Abstract

Skin is the largest organ in the human body, and the interplay between the environment factors and human skin leads to some skin diseases, such as acne, psoriasis, and atopic dermatitis. As the first line of human immune defense, skin plays significant roles in human health via preventing the invasion of pathogens that is heavily influenced by the skin microbiota. Despite being a challenging niche for microbes, human skin is colonized by diverse commensal microorganisms that shape the skin environment. The skin microbiota can affect human health, and its imbalance and dysbiosis contribute to the skin diseases. This review focuses on the advances in our understanding of skin microbiota and its interaction with human skin. Moreover, the potential roles of microbiota in skin health and diseases are described, and some key species are highlighted. The prevention, diagnosis and treatment strategies for microbe-related skin diseases, such as healthy diets, lifestyles, probiotics and prebiotics, are discussed. Strategies for modulation of skin microbiota using synthetic biology are discussed as an interesting venue for optimization of the skin-microbiota interactions. In summary, this review provides insights into human skin microbiota recovery, the interactions between human skin microbiota and diseases, and the strategies for engineering/rebuilding human skin microbiota.

## Introduction

Human skin, the body’s largest and most exposed organ, functions as a physical barrier; It is not only blocking the entry of pathogens from the environment, but also providing a large-scale ecological niche to an enormous variety of microbes [1]. The biochemical conditions of the skin are stringent, such as low pH, high salinity, exsiccosis and extensive exposure to the environmental factors. Nevertheless, many microbial species successfully colonize the skin, including bacteria, fungi, viruses (especially bacteriophages) [2]. The composition of the human skin microbiome is determined by the genetics, environmental factors, and the local microenvironment [3, 4]. Thus, the human skin microbiota varies from one body part to another, and can be very different among persons of different race, age, sex and state of health [5]. The skin microbiomes are ecosystems of diverse microorganisms that interact with the human body, including host epithelial and immune cells, as well as with other microorganisms sharing the same niche [6]. Usually, the interactions between skin microbiome and hosts are mutually reinforced. The hosts provide microbiome with ‘home’ and ‘food’, while the microbiome ‘guards’ the hosts against pathogen invasion and ‘educates’ the immune system. However, the human skin microbiome can be pathogenic, and this is closely associated with host homeostasis [7].

Some skin diseases are associated with pathogens, such as acne vulgaris [8–14], psoriasis [15–19], atopic dermatitis [20, 21], chronic wounds [22] (Table 1). Information about causal links between these diseases and key species of human microbiota is limited. With the rapid development of sequencing technologies, especially next-generation sequencing techniques [23] and long-read sequencing technologies [24], some pathogens from the human microbiota have been identified. In such cases, species-level information is not enough to identify pathogenic strains, and strain-level information is required. For example, some *Staphylococcus epidermidis* and *Propionibacterium acnes* (Current name is *Cutibacterium acnes*) strains contribute to acne and other skin diseases, while several other strains of the same species help to promote skin health by inhibiting the growth and invasion of pathogens [10]. Based on the identified microbes and their microbiological pathogenesis, insights into molecular and immunological mechanisms of microbiome-host interaction are essential.

**Table 1:** Skin microbiome and associated diseases

Disease type	Key Points	Major findings	References
Acne vulgaris	*P. acnes*	Although the relative abundances of *P. acnes* were similar, certain strains were highly associated with acne and healthy skin	Sorel Fitz-Gibbon et al. [8]
	*S. epidermidis*	*S. epidermidis* mediates fermentation to inhibit the growth of *P. acnes,* which can be implications of probiotics in acne vulgaris	Yanhan Wang et al. [9]
	*S. epidermidis* & *P. acnes*	*S. epidermidis* and *P. acnes* are thought to contribute to the disease, but they are also known to promote health by inhibiting the growth and invasion of pathogens	Alan M. O’Neill et al. [10]
	Dysbiosis & Balance	The mere presence of disease-associated strains, as well as the balance between metagenomic elements shapes the overall virulence property of the skin microbiota. Dysbiosis is the process leading to a disturbed skin barrier and disequilibrium of the cutaneous microbiome	Emma Barnard et al. [11], B. Dreno [12], Chun-xi LI et al. [13]
	Androgen hormone activity	Increases sebum production inside the pilosebaceous follicle, adjusting the environment for *P. acnes* which triggers inflammation	M. A. Rocha et al. [14]
Psoriasis	Diversity & Stability	Psoriasis induces physiological changes both at the lesion site and at the systemic lever, with increased diversity and reduced stability compared to the healthy skin microbiome	Alexander V Alekseyenko et al. [15], Daniel J. Lewis et al. [16]
	Skin microbiome	Increased abundance of *Corynebacterium, Staphylococcus*, and *Streptococcus*, and decreased abundance of *Malassezia, Propionibacterium, Cutibacterium* genera versus controls	Di Yan et al. [17], Hsin-Wen Chang et al. [18]
	Gut microbiome	The gut microbiome composition in psoriasis patients has been altered markedly, and the ratio of *Firmicutes* and *Bacteroidetes* was perturbed in psoriatic individuals compared to healthy controls	Xinyue Zhang et al. [19], Di Yan et al. [17]
Atopic dermatitis	*S. aureus*	AD has long been associated with *S. aureus* skin colonization or infection, and increases in *Streptococcus, Propionibacterium*, and *Corynebacterium* species were observed following therapy	Heidi H. Kong et al. [21], Tetsuro Kobayashi et al. [20]
Chronic wound	*S. aureus* & *P. aeruginosa*	*S. aureus* and *Pseudomonas aeruginosa* are the most common bacteria isolated from chronic wounds	Raffaele Serra et al. [22]
Skin and soft tissue infection	*Cutibacterium acne*	*C. acnes* has the potential to directly and indirectly cause inflammation and tissue damage	Laurice Floweis et al. [6]

In the future, treatment of skin diseases should not be limited only to antibiotics, topical corticosteroids, laser therapy, or other traditional strategies. Modulation of human skin and gut microbiota with healthy diet and other strategies should be considered as well. Similar to fecal microbiota transplantation to regulate gut microbiota, combinations of probiotics, skin microbiota transplantation and other developing strategies are promising venues for skin disease treatment. Building synthetic microbiota with defined and controllable properties has been applied to evaluate the principle of skin microbial interactions and dynamics [25, 26]. Engineering and rebuilding of skin microbiota could become a powerful tool to help skin diseases treatment in the future.

## Interactions between microbiota and human skin

**Fig. 1:**
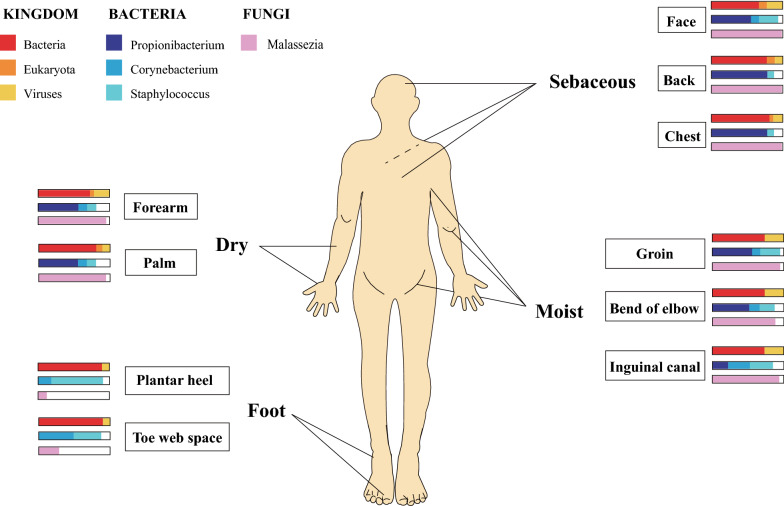
Distribution of microorganisms in human skin. Human skin area can be divided into four microenvironments, including dry (forearm and palm), sebaceous (face, back and chest), moist (groin, bend of elbow, and inguinal canal) and foot (plantar heel and toe web space). The relative abundance of viral, bacterial, and fungal components of corresponding skin microbiota are indicated. Bar charts represent relative abundance of microorganism distributed in the human skin, and the white spare in the bar charts represents other bacterial or fungal categories except the described microbes in the bar chart. The front of the human body is shown

Human skin tissue surface covers approximately 1.8 m^2^. Together with hair follicles, sebaceous glands and other associated appendages, human skin provides a habitat for > 10^10^ microbes, with 1 million microbes present per 1 cm^2^ [27, 28]. Based on the key conditions and the composition of skin microbiota, human skin can be divided into four types of environments: dry, moist, sebaceous (oil) and foot (Fig. 1) [1]. The diversity and abundance of skin microbiota in the state of health and disease are very different [6]. Most skin diseases are proved to be associated with the dysbiosis and imbalance of the skin microbiome [11, 15, 17]. Some strains and their key metabolites may be the biomarkers for diagnosis or therapeutic targets of skin diseases. Studying the interactions between the microbiota and the human skin (including the normal skin microbiota), the factors that disturb the skin microbiota, and the microbial roles in skin wound recovery, is of great interest.

### Colonization and dynamics of skin microbiota

Microorganisms that colonize the skin include bacteria, eukaryotes and viruses, and their distribution critically depends on the environmental conditions on the skin surface (Fig. 1). In general, people without skin diseases colonized with similar skin microbiota. At the kingdom level, bacteria are the most dominant. At the genus level, *Propionibacterium*, *Corynebacterium* and *Staphylococcus* represent the three most dominant microbes in the skin microbiota, each contributing positively to human health [29]. Since sebaceous glands secrete lipid-rich sebum, sebaceous sites are dominated by *P. acnes* and other lipophilic *Propionibacterium* species [30]. The *Staphylococcus*, *Corynebacterium*, and other humidity-loving species are abundant in moist areas [31, 32]. Among fungi [33], *Malassezia* species dominates the core-body and arm sites. Foot sites, the major sites of fungal infection, harbor complex fungal communities composed of *Malassezia*, *Aspergillus*, *Cryptococcus*, *Rhodotorula*, *Epicoccum* and other species.

Like the gut microbiome, the skin microbiome is a dynamic system. At the age of three, human gut microbiota typically converges toward an adult-like profile with dramatic transient shifts [34]. By contrast, the skin microbiota undergoes two dramatical changing stages. The first stage is mainly determined by the delivery mode. The skin microbiota of babies delivered vaginally matures earlier than that of babies delivered by cesarean section. Specifically, the alpha diversity of the skin microbiota of babies delivered with cesarean section is lower than that of babies that are vaginally delivered [35]. High levels of *Propionibacterium* and *Streptococcus* species were found in babies delivered with cesarean section, while high level of *Lactobacillus* species was found in babies that were vaginally delivered, presumably derived from the mother’s vagina. After birth, the skin environment undergoes dynamic structural and functional changes, including shifts in pH, water content, trans-epidermal water loss, and sebum production, all of which may influence the maturation of the skin microbiota [36]. The second dramatical change happens at adolescence stage. During puberty and sexual maturation, sebum secretion is exuberant, which supports extensive proliferation of lipophilic bacteria in the skin microbiota.

After these two phases, healthy adults maintain skin microbiota in a dynamic balance, despite the skin microbiota exposure to the environment and other individuals [3]. In fact, skin microbiota undergoes small changes during daily life due to the alteration of host biology and exposure to different environments [4, 37]. Personal care products induce highly individual skin microbiome responses, including alterations in steroid and pheromone levels, dynamics of bacterial and archaeal  structure [38]. Many skin care products contain plant-derived extracts which have antimicrobial activities [39], and the antimicrobial extracts may provide a selection pressure, leading to enrichment of resistant strains [40].

### The interactions between healthy skin and microbiota

**Fig. 2:**
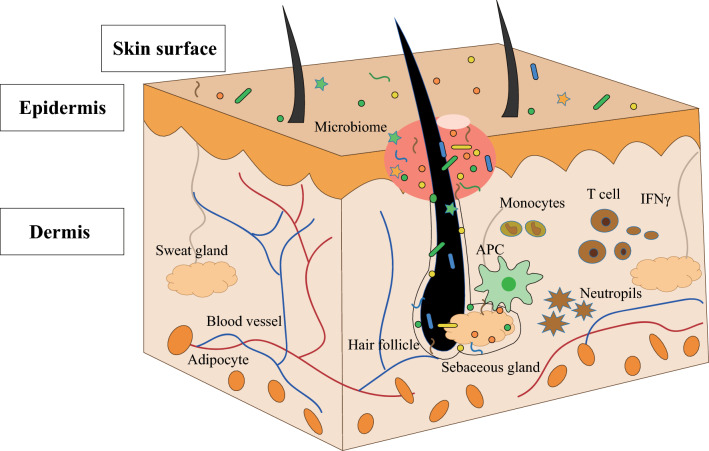
Skin structure and pathogenesis of acne. The skin structure consists of epidermis and dermis. On the skin surface, there are many hair pores, and numerous microorganisms attached. Deep in the dermis, the structure is complex, and it is composed of blood vessels, sweat glands, sebaceous glands, adipocytes, hair follicles, and immune cells. The antigen-presenting cells (APC) identify the abnormalities of attached microbes and secrete lipids, presenting a signal to the T lymphocytes (T cell), which leads to the secretion of inflammatory cytokines, such as INF γ. This leads to eradication of microorganisms by recruited neutrophil and monocytes, but contributes to the redness and formation of acne in the epidermis

Skin is composed of the surface, epidermis, and dermis (Fig. 2). Numerous microorganisms, resident or transient, colonize the surface, and they use cell debris, sebum, and mineral salts in sweat as nutrients [6]. Compared with the gut milieu, it is more difficult for the environmental microorganisms to colonize the skin. Moreover, some potential antibacterial molecules, such as natural antibiotics released by pioneering microorganisms, free fatty acids and antimicrobial peptides (AMPs) that symbiotic microorganisms secrete, can function as a protective skin barrier [29]. *Staphylococcus* strains are predominantly present at the skin surface, as they are tolerant of high salt concentration and may even utilize sweat components as nutrients. Some of them co-evolve to exchange mutual benefits with the host [41]. On the skin surface, hairs grow from the skin pores originating from hair follicles. Sebaceous glands are situated at the end of each hair follicle, providing lubrication for the hair follicle. Lipids secreted by sebaceous glands can serve as a source of nutrition for microbial growth. Hair follicle and sebaceous gland form a relatively anaerobic environment, which recruits anaerobic microbes. The microbiome of the deeper layers was described as the host indigenous microbiome, which commonly contains few microorganisms [42].

The diverse microbial species in the skin environments promote immune tolerance [43], trigger pro-inflammatory responses, and help to maintain skin health. In parallel, the host provides nutrients to the microbes and thus shapes the composition of the microbiota. *S. epidermidis* provides a good example of a close association between microbiota and the host. *S. epidermidis* is common on healthy human skin, and it is believed to be a benign microbe. Certain *S. epidermidis* strains have protective effects, achieved by secreting specific chemicals. In addition, *S. epidermis* can promote wound repair, enhance skin immunity, and inhibit pathogen infection [44]. This microbiota-host interaction contributes to the stability of microbiota and skin integrity. Some other species, such as *Roseomonas mucosa* and *Malasecia* species, can modulate keratinocytes and host immune responses in an environment-dependent manner. *P. acnes,* an anaerobic bacterium, is one of the most common and essential symbiotic bacteria of human skin [8]. *P. acnes* metabolizes sebum secretions into fatty acid for survival [45] and helps to maintain the acidic pH of the skin, which provides suitable acidic environment for specific microorganisms [46]. Skin microbiota is a collection of microorganisms that are interacting heavily among themselves, and with our skin. Thus, understanding and maintaining the delicate balance between skin and microbiota are essential steps to give insight into the mechanisms responsible for maintaining healthy skin [47].

### Roles of skin microbiota in acne

Acne vulgaris is a chronic inflammatory skin disease, and it is prevalent among teenagers. It affects approximately 85% of young persons, of which 15–20% of cases are severe [48, 49]. In addition to the formation of permanent scars, acne blocks and damages the hair follicles of adolescents. The acne is characterized by features, such as pimples, pustules and nodular cystic lesions, caused by bacteria that enter hair follicles [50, 51]. The occurrence of acne has a negative impact on the physical and psychological health of teenagers; it results in various inconveniences and feeling of inferiority [52–54]. Generally, the sebaceous gland, *P. acnes* and follicular keratinocytes are considered as the three key factors involved in the development of acne. Firstly, the excessive production of sebum from sebaceous glands blocks the hair follicles [55], leading to a relatively enclosed and anaerobic microenvironment with inflammation. This supports excessive colonization by *P. acnes*, where the increase of androgen and sebum provides a suitable environment for the growth of *P. acnes* inside hair follicles [14, 56]. Moreover, some *Malassezia* species have also been identified as the cause of refractory acne [57, 58].

*S. epidermidis*, sometimes been regarded as probiotic skin bacteria, can perform glycerol fermentation to produce short-chain fatty acids (SCFAs) which have antimicrobial activities to suppress the growth of *P. acnes* [9]. The incidence of acne is associated with disorder of the skin microbiota [11, 13]. Concretely, acne might be the result of an unbalanced state between *P. acnes* and *S. epidermidis* [59]. The microbiota of acne lesions is diverse at different zones of the body [60]. With the help of omics technologies, the incidence of acne was identified to be associated with some specific *P. acnes* and *S. epidermidis* strains in the skin cuticle [12, 13]. Therefore, a specific *S. epidermidis* strain and other strains in the skin microbiota of the acne can be potential biomarkers to predict the development of acne. Moreover, they can be the targets for accurate diagnosis and treatment of acne. The skin microbiome of patients with grade 1–3 acne was similar. However, patients with grade 4 acne showed a significantly different skin microbiome, including increased alpha diversity and overall increased presence of Gram-negative bacteria [13].

The knowledge of pathophysiology of skin disorder is limited. Host-microbiome interactions, which affect both innate and adaptive immune homeostasis, appear to be an essential factor in acne disease [10]. Besides, acne is mediated by immunity [61], as well as affected by genetics, diet and hygiene factors [54, 62–64]. At present, antibiotics were used to inhibit the growth of *P. acnes* [65]. As antibiotic resistance is becoming a growing concern in the clinical practice, it is critical to understand the skin microbiome associated with acne and find other strategies for acne treatment.

### Roles of skin microbiota in psoriasis, atopic dermatitis, rosacea and other skin diseases

Psoriasis is a common cutaneous disease with multifactorial etiology including genetic and non-genetic factors like diet, drugs, smoking, infection, and mental stress [66]. The pathogenesis of psoriasis is thought to be driven by the interactions between innate immune cells, adaptive immune cells and keratinocytes, in a process mediated by cytokines (including interleukins IL-6, IL-17 and IL-22, interferon and tumor necrosis factor) and other signaling molecules [67]. Significant skin and gut dysbiosis among patients were found to be associated with psoriasis [68]. In psoriasis-affected skin, the alpha diversity of the microbiota is found to be decreased [69]. The microbiota of the psoriasis-affected and immediately adjacent skin was similar. Specially, psoriasis lesioned skin was linked to the increase of Firmicutes, Bacteroidetes and *Streptococcus* and decrease of Actinobacteria and *Propionibacterium* [17, 70]. *Xanthomonadaceae*, assigned to be Proteobacteria and known to be keratolytic, was associated with the clinical improvement after a 3-week balneotherapy treatment [71]. Orally administered probiotics have a positive influence on the course of psoriasis [72]. Therefore, it is possible to develop accurate molecular signatures for the diagnosis of psoriasis from skin microbiome data [73]. Strain-level analyses pointed to psoriatic niche-specific strain adaptation or selection, through revealing strain heterogeneity colonization and functional variability linked with psoriasis [74].

Several different but interdependent factors might lead to atopic dermatitis (AD). The impaired barrier function of AD patients’ skin is very different from healthy skin [75]. The dysbiosis of the skin microbiota was associated with increased colonization of pathogens and a decrease in numbers of beneficial commensals [76, 77]. While the role of dysbiosis in the pathogenesis of atopic dermatitis is unclear, AD patients generally have low-diversity skin microbiota, with predominance of *S. aureus* [20]. In some situations, overgrowth of *S. aureus* precedes the development of AD [78]. In another study, One-year old infants with AD were not colonized with *S. aureus* before developing AD symptoms [77], therefore, investigation of association between abundance of *Staphylococci* and AD symptoms is of great interest.

The involvement of skin microbiota in rosacea has not been extensively studied, as the main focus has remained on *Demodex* mites [79]. These ectoparasites are improbable to be the only agent involved in the progression of rosacea. Though antibiotics are effective in treating most rosacea patients [80], it has no effects on *Demodex*, thus, microbes may be an important pathogenic factor [81]. In many clinical cases, systemic antibiotics are extensively used to control the pustules and papules of rosacea. Comparison of the skin microbiota in rosacea before and after taking oral antibiotics showed that naturally occurring *S. aureus* and *Corynebacterium bovis* colonization engendered inflammation in eczematous dermatitis [20].

### The host barrier and microbial infection

If the skin barrier is intact, it is normally difficult for the pathogens to invade. Chronic leg ulcers and many other chronic wounds affect 1–2% of the population, and cause increased morbidity and health costs [22]. Skin microbiota, especially specific pathogens, contribute to microbial infection of chronic wounds. *S. aureus* is often the cause of atopic dermatitis (AD) [82], although *S. aureus* is at other occasions just a commensal in the skin microbiome [83]. As *S. aureus* has antibiotic resistance genes, it can result in severe skin and soft tissue infection [84]. Moreover, *S. aureus* strains are highly diverse, and different patients may be colonized by different *S. aureus* strains. In a cohort study of diabetic foot ulcers, *S. aureus* was significantly enriched in non-healing wounds, and some *S. aureus* strains were associated with high morbidity [85]. *R. mucosa* depressed the growth of *S. aureus* in a murine model and in human skin via promoting production of IL-6 [86], showing the possibility to recover novel strains in the skin microbiome and use them to ameliorate dermatological disease.

*S. aureus* and *P. aeruginosa* are the most common bacteria isolated from chronic wounds [22]. They can express surface proteins and virulence factors to inhibit and decelerate wound healing. The co-infection of *S. aureus* and *P. aeruginosa* is more virulent than that of single infection [22]. The *S. aureus* strains can weaken skin barrier and activate deleterious host immune reactions [4]. It is known that dysbiosis of human skin microbiota could trigger immune dysregulation and subsequently an inflammatory response. Thus, human skin microbiota plays a key role in clinical manifestations [87].

### Gut-brain-skin axis

The gut microbiome influences skin and other distant organ systems [88]. The relationships of gut microbiome with distant organs are conceptualized as the gut–brain axis, gut–lung axis, gut–skin axis and other gut-X axes [89, 90]. The skin and gut barrier are highly similar, and they share many features [91]. Both gut and skin surfaces are covered by epithelial cells (ECs), and they contact with the exogenous environment directly [92]. With high cellular turnover rate, the adherence and infection to the gut and skin by microbes are difficult [93]. The association between skin and gut is mediated by the host immune system [94].Normally, allergic food ingredients impair the intestinal barrier and lead to food allergies, including gut and skin symptoms [95].

There is bidirectional link between gut and skin dysbiosis, and the gut microbiota dysbiosis is the response to the pathophysiology of multiple inflammatory skin diseases [96]. The composition of gut microbiota in psoriasis patients was found to vary significantly, and the Firmicutes and Bacteroidetes ratios of persons with psoriasis differ from healthy ones [17, 19]. Skin exposure to ultraviolet B light (UVB) can enhance serum vitamin D levels indirectly, which can be correlated to an increase in alpha and beta diversities of Proteobacteria phylum in the gut microbiota [97]. Moreover, gastrointestinal disorders are associated with certain dermatoses, and 7–11% of patients with IBD suffer from psoriasis [98]. Augmenting or repairing a leaky gut barrier is often applied as adjuvant therapy in the treatment of inflammatory skin diseases, which helps to increase the efficacy of standard dermatotherapy.

In the future, the skin disease treatment strategy using gut microbiota might be realized by modulation of the gut microbiota using dietary agents or selected natural/synthetic microbiota [99]. The gut–skin axis was proposed to be an integral part of the gut–brain–skin axis [100]. For example, chronic wound conditions and depression share some common pathologic features, such as altered microbiome and dysregulated inflammation. It is believed that intestine and skin can be seen as one system, and the gut-skin axis should be applied in the treatment of skin diseases. Traditional Chinese medicine (TCM) has clinical experience on the modulation of the intestinal tract and skin diseases. Nowadays, the gut-skin axis evidence is based on limited cohort studies, and a multi-systematic study with a large cohort would provide better insights into the skin microbiota and their relationships with gut microbiota [101]. This approach of studying the gut-skin axis would highlight and reveal mechanisms of action of TCM.

## Strategies for skin diseases treatment

**Fig. 3:**
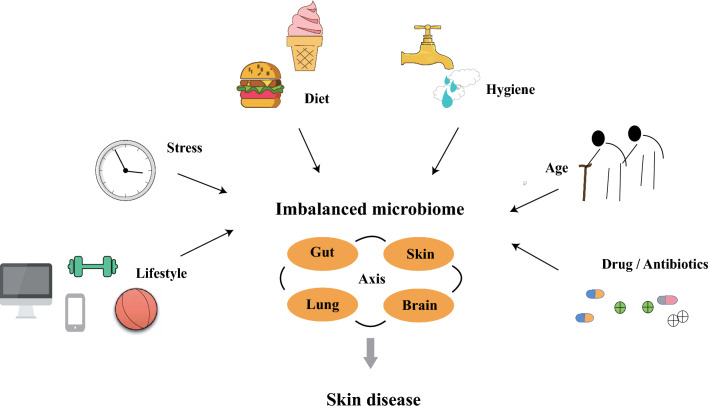
Factors associated with skin diseases. Lifestyle, stress, diet, hygiene, age, and drug (antibiotics) application are closely related to skin diseases through the links between gut, skin, lung and brain axis

Unbalanced microbiota and specific strains can cause or contribute to skin diseases. Several lifestyles are associated with skin health (Fig. 3). Exercising is beneficial to maintaining healthy skin [102]. Although regular exercise can protect the skin from free radicals, training with extreme intensity or duration or lack of training may mediate oxidative stress and contribute to skin carcinogenesis [103]. Living in a polluted environment may reduce skin moisture, increase the rate of sebum excretion, and tends to exacerbate the symptoms of chronic inflammatory skin diseases [104]. The ‘‘hygiene hypothesis’’ illustrates that lack of exposure to a full range of microbiota during childhood may lead to failure in “training” the immune system [105]. This is proposed to decrease the resistance to microbial pathogens and increase vulnerability to infection and other ailments. AD and other skin atopic diseases might be related to the excessively clean (abiotic) environment [77].

### Traditional treatments for acne and other skin diseases

Treating inflammatory or mixed acne with topical antibiotics has been prevalent for over 40 years [106]. In addition to antimicrobial effects targeting *P. acnes,* oral antibiotics have anti-inflammatory effects via host immune responses [107]. Topical ofloxacin possess potent antimicrobial activity against the *Propionibacterium* and *Staphylococci* strains isolated from acne patients, making it an effective therapeutic drug for acne vulgaris [108]. Based on anti-inflammatory properties, topical retinoids are effective in the treatment of inflammatory lesions [109]. Several treatment guidelines and expert consensus documents suggest that macrolides, clindamycin and tetracyclines are recommended as the first-line therapy drugs in acute inflammatory phase of acne [110, 111].

Nowadays, nucleic acids (NAs) play significant roles in the treatment of several diseases. Topical NAs or NAs-based delivery of drugs have special advantages in skin disease treatment, due to the efficient NAs transfer and direct targeting to the skin disease sites [112]. Multitudes of NA-based therapeutics, including genes, siRNA, aptamers, antisense oligodeoxynucleotides (ODNs), and CpG oligonucleotides, have been applied for disease treatment [112]. Transdermal drug delivery (TDDS) has been highly explored, due to the advantages of special tissue targeting, improved drug release, avoiding the presystemic metabolism, high tolerance in patients, and less toxic to the patient’s liver [113]. Currently, a variety of nanoparticles and nanoemulsions are applied in TDDS to treat psoriasis, wound healing, melanoma and other skin diseases [114]. Low-level laser (light) therapy (LLLT) is a fast-developing technology for the treatment of diseases that require relief of pain and inflammation, function restoration, or healing stimulation. LLLT has positive effects on wrinkles, acne scars, hypertrophic scars, healing of burns, psoriasis and acne, and other inflammatory diseases [115]. LLLT can reduce UV damages both as a treatment strategy and as a prophylaxis. Its non-invasive characteristics and almost complete absence of side-effects warrant further exploration and application in dermatology.

### Healthy diets and lifestyles affect skin microbiota

Despite strong lay beliefs that diet is not a dominant factor in acne, increased self-reported cases show that adolescent acne is associated with frequent consumption of milk and milk-containing foods (instant breakfast drink, cottage cheese, and cream cheese), and nonfat portion of milk seems to have a stronger association with acne than that of whole or low-fat milk [116, 117]. The skimmed milk contains hormones and bioactive molecules, and it may have an acne stimulating effect due to androgens, progesterone, and insulin growth factor-1 (IGF-1) [116]. Western diets, which are typically high-glycemic-load (HGL) diets, can elevate IGF-1 and blood insulin levels chronically or acutely, which leads to increased sebum production and even acne [118]. There are positive correlations between acne severity and high glycemic load foods [64]. As a result, dermatologists usually suggest that acne patients avoid high glycemic index foods [64].

Personal hygiene is closely related to acne, and too much washing may actually worsen the condition. Generally, washing twice daily appears to be the best recommendation [119]. Two independent groups of studies among young students suggested a correlation between acne severity and stress levels during examination periods [120, 121]. Regular exercise (4 h per week high-intensity aerobic exercise) reduced the thinning of the stratum corneum in patients compared with that in sedentary controls (1 h per week high-intensity aerobic exercise) [122]. The expression level of Pgc-1 (the master regulator of mitochondrial biogenesis) increased after exercise [123], which can decrease aging of skin cells. Additionally, vigorous exercise, especially calisthenics and aerobic exercise, was independently linked to reduced risk of psoriasis in US women [124]. Therefore, proper diet, cleaning, moisturizing and exercise should be applied together with other adopted skin disease therapeutic strategies [14].

### Designed mixture of probiotics and prebiotics

The first generation of microbiota therapies consisted of probiotics and prebiotics. They are designed to maintain, restore and optimize the skin microbiota in different ways. Topical applications of probiotics are beneficial for cutaneous immune responses and eliminate pathogens by enhancing the skin natural defense barriers or producing antimicrobial peptides [125]. Prebiotics are non-digestible foods or metabolites degraded by the gut bacteria [126]. Prebiotics were applied in cosmetic formulations directly, in order to promote the growth and activity of beneficial skin microbiota [125]. Among the prebiotics, plants, especially traditional Chinese medical plants, can provide diverse natural products for skin microbiota. Insights into the traditional Chinese medical plants identified various bioactive components. As plant growth takes long time and extraction of the bioactive natural products is difficult, engineered yeasts were used for the efficient production of these bioactive natural products [127, 128]. Several traditional Chinese medical plant-derived natural products, such as ginsenosides, monoterpenoids, glycyrrhetinic acids, have been successfully synthesized using engineered yeasts [129, 130].

**Fig. 4:**
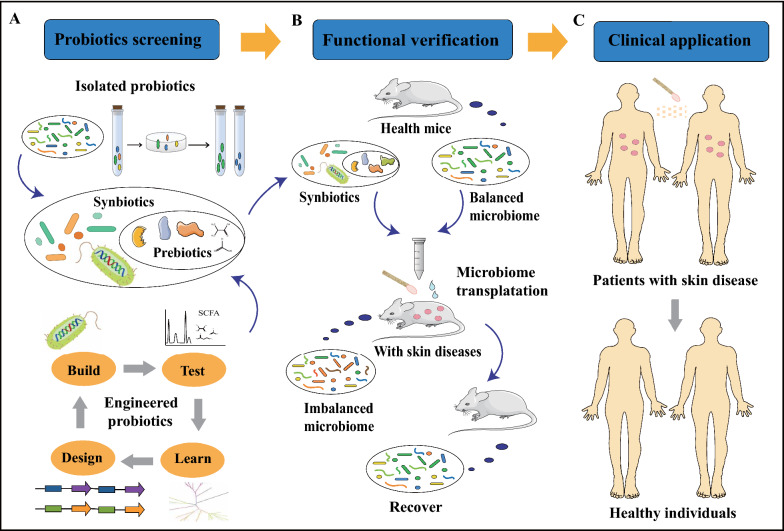
Microbiome strategies used to modulate skin microbiome in a balanced state. **A** Methods to discover/build new probiotics, prebiotics and synbiotics. Isolating and identifying bacteria from healthy gut or skin microbiome to discover new probiotics would lead to discover of functional probiotics. Probiotics with beneficial functions to skin health can be engineered or built using synthetic biology strategies. Combination of these probiotics and functional prebiotics form synbiotics. **B** Functional verification of synbiotics and healthy microbiome in vivo. Designed synbiotics (**A**) and balanced microbiome from the healthy mice to the mice with skin diseases can revert their imbalanced microbiome. **C** Microbiome transplantation to balance skin microbiota in clinical applications. Transplantation of healthy microbiome from healthy persons’ skin as a whole or specifically isolated probiotics and engineered probiotics, can revert the imbalance

Based on the gut and skin microbiome, new probiotic and prebiotic products for the treatment of multitudinous skin conditions can be developed [131] (Fig. 4A). Probiotics derived from *S. epidermidis* can help restore the naturally balanced microbiota and regulate the host’s AMP mediators [59]. Coagulase-negative *Staphylococci* (CoNS) strains are commonly distributed on the skin surfaces of healthy persons, but are rare on that of the AD patients [132]. The antimicrobial activity of CoNS species, such as *S. epidermidis* and *S. hominis*, is based on AMPs they produce. Absence of CoNS strains may lead to excessive colonization of *S. aureus* on the skin of AD patients. The AMPs were found to be strain-specific and highly potent; They can selectively kill *S. aureus* and synergize with the human AMP LL-37 [132].

Mixtures of different skin microorganisms in definite proportions can change the composition of the recipients’ skin microbiomes [133]. After sequential applications of a donor microbiome, the recipients’ microbiota becomes similar to the donors’ microbiota gradually, showing that using living bacteria to modulate skin microbiome composition is possible (Fig. 4B). Moreover, transferring probiotic solutions from facial skin microbes of healthy volunteers to the faces of acne patients can improve skin health (Fig. 4C)*.* Application of natural bacteria onto the skin can improve moisture retention and decrease skin pH [134], and it opens the possibility to develop probiotic solutions which might help the human skin revert from disease microbiota state to a healthy state.

Manipulation of skin microbiota with increased abundance of beneficial species may reduce the presence of undesirable pathogens and promote skin health directly [135]. Some specific microorganisms in the skin microbiome can reduce the colonization and overgrowth of *P. acnes* by fermenting glycerol and creating inhibition zones [136]. Clinical isolates of CoNS species residing on normal skin microbiota produce autoinducing peptides to disturb the quorum sensing system of *S. aureus*, which decreases phenol-soluble modulin (PSM) expression and abolishes biofilm attachment and regrowth. A clinical isolate of *S. hominis* synthesizes an autoinducing peptide (SYNVCGGYF), which is a highly potent inhibitor of *S. aureus* Agr-mediated quorum sensing, and this could prevent *S. aureus*–mediated epithelial damage and inflammation on murine skin [137]. Oral probiotic interventions were investigated for clinical application in diverse diseases, but external skin commensals used to treat skin diseases are rarely reported [138]. Further discovery of probiotic functions in skin microbiota would be useful in future skin disease treatments. Synbiotics is the combination of prebiotics and probiotics. In the future, synbiotics will expand out possibilities to effectively treat skin diseases (Fig. 4).

### Engineering and rebuilding of skin microbiota

Synthetic biology strategies applied on the microbiome can lead to characterization of underlying roles of skin microbiota in disease development, and develop novel diagnostic and therapeutic strategies for skin diseases. Different omics techniques: metagenome, metatranscriptome, metaproteome, and metabolome, can give systematic and global view of the skin microbiota. Biosensors, memory arrays, engineered bacteria, and other tools can be sued to rewire the microbiome [139]. Quick sensing of a stimulus in situ that can immediately trigger a precise therapeutic strategy would rebalance the dysbiosis of the skin microbiome and contribute to curing the skin diseases [139].

The knowledge gap in our understanding of the link between the skin microbiota and skin diseases is still substantial, and the application of probiotics in skin microbiota modulation has not been realized yet. It remains a challenge to reach a conclusion about the proper dose and formulation of probiotics [140]. Moreover, identification of skin probiotics is difficult, due to the genetic differences between strains [141]. Therefore, development of engineered strains with desired beneficial functions is necessary, and synthetic biology strategies could provide gene editing and other tools for strain reprograming (Fig. 4A). The engineered probiotics and the functional prebiotics they produce could be applied for the treatment of skin disease directly. The gene editing tool can be delivered with phages to remove designated pathogenic strains [142], showing the potential for future skin disease treatment based on targeted microbiota modulation. In summary, advanced synthetic biology enables new approaches to design and reprogram multispecies microbiota, which presents an exciting opportunity to rationally engineer or rebuild skin microbiota for skin disease treatment [26, 139].

## Future perspectives and conclusion

Skin microbiota plays essential roles in skin disease occurrence and development. Therefore, modulation of skin microbiota is one of the best strategies used for skin disease treatment. At present, diverse microbiota modulation strategies are available, including prebiotics and probiotics. Nowadays, some skin care products contain herbal ingredients and other prebiotics, in order to maintain skin healthy. Though oral probiotics products are regularly used to improve intestinal flora via relieving indigestion and intestinal inflammation, few probiotics are applied in the modulation of skin microbiota. This is mainly due to the fact that the available information on skin microbiota is limited. The omics technologies can give insights into the interaction between skin microbiota and skin diseases and identify microbial markers for the diagnosis and treatment of skin diseases. Species-level and strain-level information of the microbiota related with diverse skin diseases need to be revealed. Clinical isolation of pathogenic strains and probiotics will help reveal the causal relationship between skin microbiota and skin diseases.

In the future, in situ monitoring of skin microbiota at the disease sites and identifying the pathogenic strains will provide crucial preliminary information for skin disease treatment. Further precise treatment strategies, such as addition of prebiotics and probiotics, engineering or rebuilding synthetic microbiota with desired characteristics, will be applied in clinics. The future treatment pipeline will comprise the following steps: recovering personal skin microbiota, isolating the key pathogenic bacteria, revealing pathogenic mechanisms, and developing effective prebiotics/probiotics agents to counter specific pathogens. The skin microbiota modulation strategy will not only relieve the occurrence and development of skin diseases, but also improve the appearance and maintain physical and mental health.

## Data Availability

Not Applicable.
